# Heritability of REM sleep neurophysiology in adolescence

**DOI:** 10.1038/s41398-022-02106-6

**Published:** 2022-09-21

**Authors:** Andjela Markovic, Michael Kaess, Leila Tarokh

**Affiliations:** 1 University Hospital of Child and Adolescent Psychiatry and Psychotherapy, University of Bern, Bern, Switzerland; 2 Department of Child and Adolescent Psychiatry, Center for Psychosocial Medicine, University Hospital Heidelberg, Heidelberg, Germany; 3 Translational Research Center, University Hospital of Psychiatry and Psychotherapy, University of Bern, Bern, Switzerland

**Keywords:** Clinical genetics, Predictive markers

## Abstract

Alterations of rapid eye movement (REM) sleep have long been observed in patients with psychiatric disorders and proposed as an endophenotype—a link between behavior and genes. Recent experimental work has shown that REM sleep plays an important role in the emotional processing of memories, emotion regulation, and is altered in the presence of stress, suggesting a mechanism by which REM sleep may impact psychiatric illness. REM sleep shows a developmental progression and increases during adolescence—a period of rapid maturation of the emotional centers of the brain. This study uses a behavioral genetics approach to understand the relative contribution of genes, shared environmental and unique environmental factors to REM sleep neurophysiology in adolescents. Eighteen monozygotic (MZ; *n* = 36; 18 females) and 12 dizygotic (DZ; *n* = 24; 12 females) same-sex twin pairs (mean age = 12.46; SD = 1.36) underwent whole-night high-density sleep EEG recordings. We find a significant genetic contribution to REM sleep EEG power across frequency bands, explaining, on average, between 75 to 88% of the variance in power, dependent on the frequency band. In the lower frequency bands between delta and sigma, however, we find an additional impact of shared environmental factors over prescribed regions. We hypothesize that these regions may reflect the contribution of familial and environmental stress shared amongst the twins. The observed strong genetic contribution to REM sleep EEG power in early adolescence establish REM sleep neurophysiology as a potentially strong endophenotype, even in adolescence—a period marked by significant brain maturation.

## Introduction

After rapid eye movement (REM) sleep was discovered by Nathaniel Kleitman in 1953, REM sleep has been a major focus of sleep research. In addition to rapid eye movements, REM sleep is characterized by reduced muscle tone and low-amplitude EEG activity of mixed frequencies. This oscillatory activity is similar to waking, which is why REM sleep has also been called paradoxical sleep. While the function of REM sleep remains elusive, recent work has highlighted the role of REM sleep in memory processing [1] and emotional functioning (reviewed in Goldstein and Walker, 2014) suggesting a significant role of REM sleep in everyday functioning. Goldstein and Walker [2] proposed the “REM sleep emotional homeostasis hypothesis” positing that not only are affective experiences consolidated during REM sleep but also that the emotional strength of negative memories is reduced, renormalizing the brain’s sensitivity to emotional stimuli. This hypothesis is supported by observations of altered emotional responses after sleep disruption [3] and restriction [4], showing an amplified reaction of the amygdala to negative emotional stimuli linked to the duration of REM sleep.

Furthermore, the strength of EEG oscillatory activity in the theta range (4–7 Hz) during REM sleep has been correlated with sleep-dependent consolidation of emotional memories [5, 6], suggesting that brain activity during REM sleep as measured via the EEG can provide insight into the neural processes achieving emotional homeostasis. Along the same lines, increased theta activity during REM sleep has been found in nightmare recallers as compared to those who do not frequently recall nightmares, increased in those who recall dreams as compared to those who do not upon awakening (Marzano et al., 2019). Furthermore, REM sleep prefrontal theta activity is correlated with the degree to which recent waking-life experiences are incorporated into dream content [7]. These findings and others [8, 9] have reinforced the notion that REM sleep prefrontal theta activity plays an important role in sleep-dependent emotional processing. Such observations in humans are in line with a well-established literature in rodents that has shown that theta oscillations in REM sleep are highly synchronized between the hippocampus, amygdala, and the neocortex and that the synchronization of these areas is important for achieving emotional recalibration during sleep [10].

Given these findings in healthy populations, it is not surprising that REM sleep has been mechanistically linked to psychiatric disorders. For example, alterations in REM sleep characteristics have been observed in disorders associated with stress and emotion dysregulation, such as posttraumatic stress disorder [11] and major depressive disorder [12]. In posttraumatic stress disorder (PTSD), decreased and fragmented REM sleep associated with increased activity of the noradrenergic system has been reported [11]. Furthermore, susceptibility to PTSD symptoms following a traumatic event has been tied to REM sleep prefrontal theta, suggesting that this may be a useful biomarker for susceptibility to stress. Similarly, increased REM sleep density has been proposed as a vulnerability marker for depression (e.g., Lauer et al. [13],) and prefrontal REM sleep theta has been shown to predict response to antidepressants in a sample of depressed adults [14]. These studies raise the possibility that REM sleep theta power over prefrontal regions may be a marker for vulnerability to psychiatric illness and one mechanism of action may be through the impact of stress on REM sleep. Indeed, in mice exposed to stress, REM sleep duration and theta power were increased [15]. In order to understand why theta power is a marker for vulnerability understanding the degree to which genetic and environmental factors contribute to this metric is an important first step.

If a genetic contribution to this measure exists, then REM sleep power may be a useful endophenotype for psychiatric disorders. Endophenotypes provide a link between genes and behavior and, as such, could aid in identifying those with a genetic vulnerability to a psychiatric disorder. In addition to being objectively measurable and associated with illness in the population, a key criterion for an endophenotype is that they are heritable. Therefore, given that REM sleep power is objectively measurable and associated with illness, in this study, we set out to examine whether the REM sleep power meets the heritability criteria for an endophenotype.

One way to address this question is through the use of a twin design. Twin studies are based on the assumption that monozygotic (MZ; identical) twins have all their genes in common, while in dizygotic (DZ; non-identical) twins, this proportion amounts to ~50 %. Therefore, if MZ twins are more similar with regards to a phenotype than DZ twins, this similarity is attributed to the greater proportion of genes shared amongst MZ as compared to DZ twins. A previous study in adults using a twin design has shown significant genetic control of REM sleep duration, while the genetic influence was not significant for REM sleep latency [16]. In this study, sleep EEG power was analyzed at one central EEG derivation, where the authors found high heritability of EEG power across all frequency bands. Given that previous studies have almost exclusively found that REM sleep theta power in prefrontal regions is associated with emotional processing and psychiatric illness, the question of the degree to which genetic and/or environmental factors impact prefrontal REM sleep theta power remains open.

Therefore, the aim of the current study was to quantify the genetic and environmental contribution to sleep EEG power across brain regions using high-density sleep EEG in adolescence. We examine adolescence because many psychiatric illnesses arise during this developmental phase [17], making a genetic measure of vulnerability to stress and psychiatric illness valuable for early detection and intervention. Furthermore, during adolescence, REM sleep undergoes profound neurophysiological changes with a marked decline in REM sleep EEG power across this phase [18, 19]. Finally, the emotional centers of the brain undergo significant maturation during adolescence and REM sleep may play a unique role in the development of these processes. Based on previous findings in adults [16], we hypothesize a strong genetic impact over central regions across frequency bands.

## Methods

### Participants

This study was based on a twin study including eighteen monozygotic (MZ; *n* = 36; 18 females) and 12 dizygotic (DZ; *n* = 24; 12 females) same-sex twin pairs (mean age = 12.46; SD = 1.36) who underwent whole-night high-density sleep EEG recordings. One participant who was part of a triplet consisting of one MZ and one DZ pair was included in both groups. Findings with regard to NREM sleep have been previously published [20–22]. No significant differences between the groups were found with regards to age, gender, or pubertal status. A questionnaire with 95% accuracy [23] filled out by the parents was used to determine zygosity. Only healthy participants born after 30 weeks of gestational age were included in the study. We obtained written assent from all participants and written consent from their parents. The study was approved by the ethics committee of the Canton of Zurich. All procedures were performed according to the Declaration of Helsinki.

### Procedures

We conducted sleep EEG recordings at families’ homes for two consecutive nights (adaptation and baseline night). Only recordings from the baseline night (second night) were included in analyses with the exception of three participants, for whom the baseline recordings were of insufficient quality, and data from the adaptation night (first night) were used. Prior to the recordings, participants complied with a sleep schedule for at least 5 days, ensuring adequate sleep of at least 9.5 h time in bed (Carskadon, 1982; Short et al., 2018). Actigraphy and sleep diaries were used to verify compliance. All procedures were repeated 6 months later (i.e., sleep schedule, adaptation, and baseline) in 14 monozygotic (MZ; *n* = 28; mean age = 13; SD = 1.3; 14 females) and 11 dizygotic (DZ; *n* = 22; mean age = 13.5; SD = 0.7; 6 females) twin pairs (five twin pairs dropped out of the study). The data from these follow-up measures are presented in the supplements in order to show the stability of our findings.

### Sleep EEG Analysis

A Geodesics EEG system (GSN300; Electrical Geodesic Inc., Eugene, OR, USA) with 64 channels (58 EEG, 2 electrooculogram, 2 electromyogram, and 2 electrocardiogram channels) was applied with a sampling rate of 1000 Hz (downsampled to 250 Hz for analysis). Channels with insufficient data quality were excluded based on visual inspection and the signals were then re-referenced to the average of all remaining derivations (average reference). Sleep recordings were scored in 30-s epochs according to Rechtschaffen and Kales [24]. Power density spectra were calculated in MATLAB (Mathworks, Natick MA, USA) for each 30-s epoch (5-s windows; Hanning window; no overlap). A semi-automated procedure detected epochs with artifacts whenever power exceeded a threshold in low (0.8–4.6 Hz) or high (20–40 Hz) frequencies [25]. We analyzed power at each derivation during REM sleep in the following frequency bands: delta (1–4.6 Hz), theta (4.8–7.8 Hz), alpha (8–10.8 Hz), sigma (11–16 Hz), beta 1 (16.2–20 Hz), beta 2 (20.2–24 Hz), gamma 1 (24.2–34 Hz), and gamma 2 (34.2–44 Hz).

### Statistical analysis

Genetic and environmental influences on EEG power during REM sleep were estimated by means of structural equation modeling (SEM) with OpenMx in R [26], controlling for age and sex. We performed a power analysis using Mx, hypothesising a large (>80%) contribution of genetic factors based on our previous work examining heritability in NREM sleep (Rusterholz et al., Journal of Neuroscience, 2018) and studies in adults (Adamcyzk et al., Trans Psych, 2015) and found that despite our small sample size we have adequate power to detect medium effect sizes. The contributions of latent factors—genes (A), environmental factors shared between twins (C), and environmental factors *unique* to each twin and measurement error (E)—were calculated based on the assumption that MZ twins have all of their genes in common, while DZ twins are approximately 50% genetically concordant. All twin pairs in our sample were raised together, and thus the latent factor E comprises those environmental factors unique to each individual as well as measurement error and is, thus, uncorrelated among both MZ and DZ twins. The contributions of A, C, and E can range from 0 to 1, with all factors summing up to 1 and represent the amount of variance explained by each factor. Typically, twin studies [27–29] apply the Akaike information criterion (AIC) to measure the goodness of fit. Therefore, when the AIC was lower for a reduced model (AE or CE), indicating a better model fit, we applied the reduced model and the value of the remaining factor was set to zero [30]. We also tested the twin assumption in the saturated model and compare AIC values for the saturated and full model using a paired *t*-test across channels for each frequency (i.e., 58 values corresponding to channels).

## Results

We found no significant differences between MZ and DZ twins with regard to any sleep stage parameter (Table 1). As previously reported, all participants were good sleepers with sleep efficiency, defined as total sleep time divided by time in bed, greater than 90%, and sleep architecture typical for this age group [21, 22].

**Table 1:** Mean and standard deviation (in parentheses) of sleep parameters for monozygotic (MZ; *n* = 36) and dizygotic (DZ; *n* = 24) twins as previously reported [21].

Sleep parameter	MZ	DZ	z-statistic
Total sleep time (min)	522.61 (±51.16)	546.12 (±37.71)	−1.84 (*p* = 0.07)
Wake after sleep onset (min)	28.71 (±29.03)	23.29 (±25.94)	0.45 (*p* = 0.65)
Sleep latency (min)	22.01 (±17.85)	18.44 (±9.71)	0.27 (*p* = 0.79)
Sleep efficiency (%)	91.03 (±5.45)	92.69 (±4.33)	−0.92 (*p* = 0.36)
REM latency (min)	112.39 (±44.95)	93.06 (±40.12)	1.91 (*p* = 0.06)
Stage 2 (%)	44.26 (±10.08)	45.10 (±8.62)	−0.24 (*p* = 0.81)
Slow wave sleep (%)	29.36 (±9.56)	27.00 (±7.81)	0.86 (*p* = 0.39)
Stage REM (%)	25.98 (±5.01)	26.95 (±6.54)	−0.39 (*p* = 0.69)

The percent values were calculated with respect to total sleep time. Sleep latency was defined as the first occurrence of stage 2 sleep following lights out. Results from a Wilcoxon rank-sum test comparing the two groups with regard to sleep parameters are shown in the last column (*z* values; *p* values in parentheses).

**Fig. 1:**
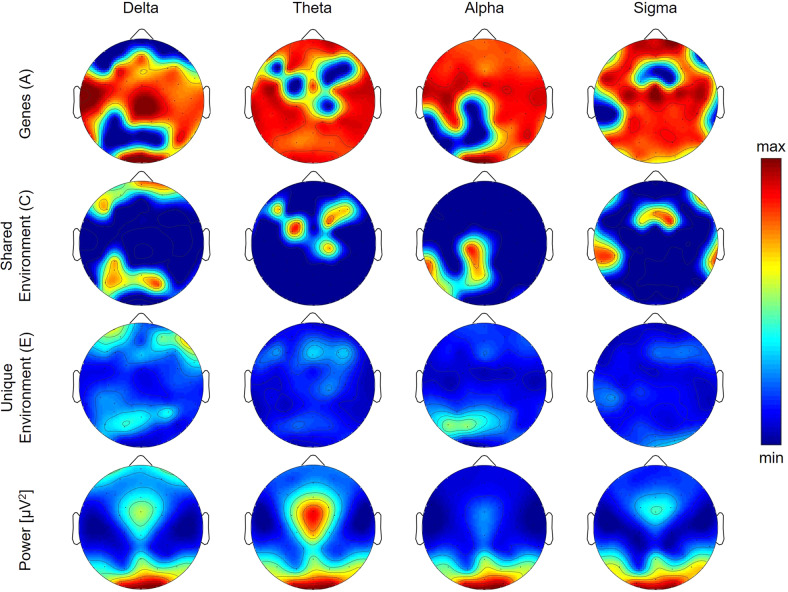
Topographic maps of genetic, shared environmental and unique environmental contribution to REM sleep Delta, Theta, Alpha and Sigma power. Top three rows show the topographic distribution of the results from structural equation modeling (SEM) for REM sleep delta to sigma bands, with the first row depicting the contribution of genetic factors (latent factor A), the second row depicting the contribution of environmental factors shared among twins (C), and the third row depicting the contribution of environmental factors unique to each twin (E). The color corresponds to the amount of variance explained by each of the factors, with warm tones representing large values (close to 1) and cool tones representing low values (close to 0). The bottom row shows the topographic distribution of power averaged across all participants, independent of zygosity.

**Fig. 2:**
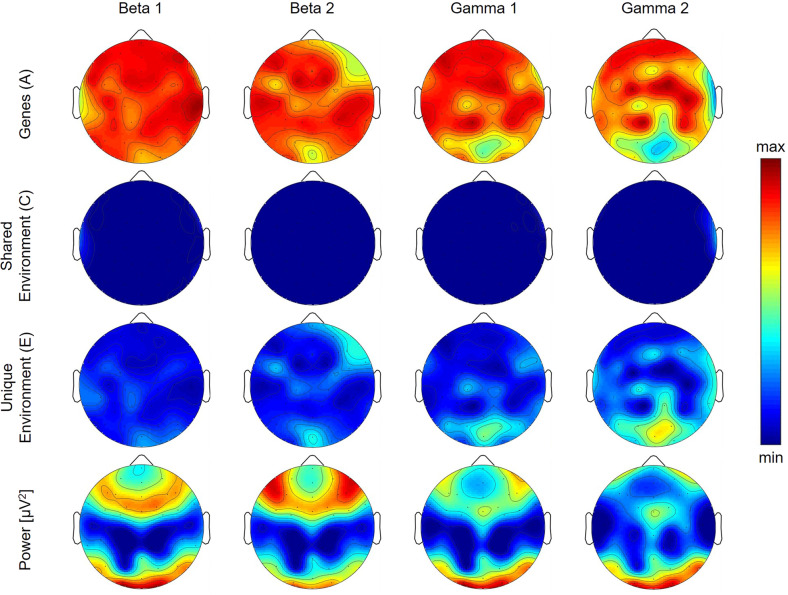
Topographic maps of genetic, shared environmental and unique environmental contribution to REM sleep Beta 1, Beta 2, Gamma 1 and Gamma 2 power. Top three rows show the topographic distribution of the results from structural equation modeling (SEM) for REM sleep beta 1 to gamma 2 bands, with the first row depicting the contribution of genetic factors (latent factor A), the second row depicting the contribution of environmental factors shared among twins (C), and the third row depicting the contribution of environmental factors unique to each twin (E). The color corresponds to the amount of variance explained by each of the factors, with warm tones representing large values (close to 1) and cool tones representing low values (close to 0). The bottom row shows the topographic distribution of power averaged across all participants, independent of zygosity.

With regard to the topographic distribution of power during REM sleep [20], REM sleep power demonstrated a strong focus over occipital regions in all examined frequency bands (last row in Figs. 1 and 2). Delta and theta bands showed an additional peak over the vertex (Fig. 1), while strong activity was observed over frontal areas in the beta bands (Fig. 2). This pattern of REM sleep power is similar to what has been reported in adolescents and is typical of this age (Markovic et al. [20]).

Examining the genetic and environmental contributors to REM sleep power results from the SEM analyses indicate a large proportion of the variance in REM sleep power across frequencies (Supplementary Table 1 and Fig. 1) is due to genetic factors. Over central and temporal regions, genetic factors accounted for more than half the variance in the delta band (35 derivations with contributions between 0.57 and 0.92, Fig. 1). For this band, we found a shared environmental impact in occipital regions (nine derivations with contributions between 0.58 and 0.83) and shared (nine derivations with contributions between 0.26 and 0.85) and unique environmental influence (11 derivations with contributions between 0.21 and 0.73) in frontal regions. In the theta band, the genetic impact was focused on frontal, temporal, and occipital regions (Fig. 1; 49 derivations with contributions between 0.66 and 0.97). We found contributions of shared environmental factors in frontal and central regions for this band (8 derivations with contributions between 0.68 and 0.95).

Genetic factors contributed to alpha power across regions (Fig. 1; 45 derivations with contributions between 0.71 and 0.96) with the exception of a centro-parietal region showing shared environmental impact (11 derivations with contributions between 0.47 and 0.86). The variance in sigma power was, for the most part, driven by genes over frontal, central, and occipital areas (Fig. 1; 44 derivations with contributions between 0.27 and 0.97), while shared environmental factors contributed to sigma power over fronto-central and temporal regions (14 derivations with contributions between 0.54 and 0.89). The estimated genetic contribution to higher frequencies (Fig. 2; beta 1 to gamma 2 bands) was regionally widespread (contributions between 0.34 and 0.99). Environmental contributions to REM sleep power in this frequency range were negligible.

We also compared the AIC values of the saturated model by performing a paired t-test. We did not find a difference between the two models for any frequency band (see Supplementary Tables 1–8 for results across frequency bands and channels).

With regards to the follow-up assessment, we found a similar pattern of results as observed in the initial assessment. Results for the follow-up assessment are shown in Supplementary Fig. 2. As apparent from this Figure, at both the initial and follow-up assessment, genetic factors are estimated to make a large contribution to sleep EEG power across frequencies, while the contribution of unique environmental factors is minimal. Furthermore, similar to the initial assessment, at follow-up, shared environmental factors contribute to power in the delta, theta, and alpha bands only. Therefore, our findings with regards to genetic and shared environmental contributors to REM sleep power were largely stable across 6 months.

## Discussion

In the present study, we examined the heritability of REM sleep EEG power during adolescence and based on estimates from the current data set, we find considerable genetic impact across brain regions and frequency bands. This finding is in line with findings in adults (Adamczyk et al. [16]), which also find that much of the variance in REM sleep EEG power over central regions and across frequencies is due to genetic factors. However, by using high-density sleep EEG, we uncover a rich topographic pattern whereby both genetic and environmental factors differentially exert influence on sleep EEG power dependent on frequency and region.

Most revealing is the influence of genetic and shared environmental factors on REM sleep theta power dependent on region. Over prefrontal regions, we find that shared environmental factors account for much of the variance in REM sleep theta power. We hypothesize that the degree of stress in the familial environment shared amongst twins may account for our observation, suggesting that previous findings of associations between prefrontal REM sleep theta power and emotional processing during sleep may be driven by shared environmental factors. To wit, in humans, a link between environmental stress and REM sleep theta activity has been reported and hypothesized to facilitate selective emotional memory consolidation [9]. Furthermore, REM sleep is known to correlate with the secretion of cortisol [2], a hormone released in response to stress [31]. Since both MZ and DZ twins in our sample shared the same stressful/non-stressful family environment, this factor may account for similarities between twin pairs and the observed shared environmental impact. Furthermore, our results put previous findings of enhanced theta power in psychiatric disorders such as PTSD and depression in a context suggesting that environmental factors early in life and not genetic factors may be responsible for the observed differences. However, since we do not directly test the association between REM sleep and psychopathology in our sample of twins, our interpretations regarding the genetic/environmental contributors to changes in REM sleep in psychiatric samples are tentative. Furthermore, shared environmental factors other than stress may underlie our findings, and future studies should examine how the environment may shape REM sleep theta power.

Similar to NREM sleep [22], power in the high frequencies (i.e., beta and gamma bands) were predominantly influenced by genes across brain regions. Considering that high frequencies are implicated in several brain disorders, such state-unspecific genetic contribution suggests that sleep EEG high-frequency activity may be a powerful endophenotype that is stable across states. For example, altered beta activity in waking has been reported in Alzheimer’s disease [32] and posttraumatic stress disorder [33]. The large genetic impact on REM sleep EEG power we estimated across frequency bands is comparable to findings in adults by Adamczyk et al. [16], who examined heritability over two central derivations (C3 and C4). For example, we also find a large genetic contribution to power over central derivations across frequencies. Because this previous study did not measure across other regions, whether the regional differences in heritability that we observe are a fundamental feature of REM sleep across the lifespan or are unique to this adolescent sample remains unknown. Future studies on adults should address this gap in the literature.

Although the role of REM sleep oscillatory activity in emotional processing in adults is becoming increasingly clear, few studies have examined this phenomenon in adolescence. What is clear is that REM sleep plays an important role in brain development early in life (reviewed in Blumberg, 2020), and recently, it has been proposed that this may also be the case during adolescence [34]. During adolescence, there is rapid maturation of the emotional centers of the brain, including the amygdala, the anterior cingulate cortex, and the prefrontal cortex—this would theoretically increase the need for REM sleep during the adolescent period. Indeed, some data suggest an increase in REM sleep during adolescence (Campbell et al., 2016). Our study adds to the existing literature showing the degree to which environmental and genetic factors shape REM sleep oscillatory activity during this developmental phase. By laying this groundwork, future studies can begin to explore whether the sleep EEG can be exploited as an endophenotype in psychiatric disorders or explore environmental factors that may shape sleep EEG power in the delta to sigma bands.

### Limitations

Several limitations of this study are important to note. REM sleep consists of both tonic and phasic components. During the phasic portion of REM sleep, which is shorter in duration than the tonic phase, eye movements are present. One limitation of the current study is that we did not distinguish between tonic and phasic REM sleep and applied no detection of rapid eye movements thus, this activity was thus included in our analyses. However, as such activity would only affect low-frequency activity at frontal derivations and is reflected in the E factor (Fig. 1), we believe that rapid eye movements do not significantly influence the remaining findings. Furthermore, our sample size was modest, as reflected in large confidence intervals for some channels/frequency bands (Supplementary Tables 1–8) and future studies should confirm our findings using larger sample sizes. Nonetheless, we were able to show that our results are stable 6 months later, which strengthens our findings and makes it unlikely that our findings are due to noise. Finally, we note the limitations inherent to twin studies, which include: (a) findings from twin studies cannot be directly generalized to the general population since participants are not randomly sampled from the population, (b) the inability to examine genes by environment interactions, and (c) the assumption that identical twins share 100% of their genetic material and that fraternal twins share 50%, which may slightly vary.

## Conclusions

Based on the sample, we estimate the high heritability of REM sleep EEG power in adolescence across brain regions and frequency bands. However, several exceptions, such as frontal theta power, indicate a considerable influence of environmental factors shared among twins. These observations suggest that environmental factors may be implicated in psychiatric disorders associated with altered REM theta power rather than genetic susceptibility. In the future, identifying such factors may uncover novel targets for health interventions.

## Supplementary information

Supplemental File

Supplementary Figure 1

Supplementary Figure 2
