# Supplementary material for: Heritability of REM sleep neurophysiology in adolescence
Source: Transl Psychiatry. 2022 Sep 21;12:399. doi: 10.1038/s41398-022-02106-6 (PMC9492899; doi:10.1038/s41398-022-02106-6)
Supplement: Supplementary file 1 — Supplemental File [file 41398_2022_2106_MOESM1_ESM.docx]

Supplementary Information

Heritability of REM Sleep Neurophysiology in Adolescence

Markovic, Kaess, and Tarokh

**Supplementary Figure 1:** In these figures genetic (A), shared environmental (C) and unique environmental/error (E) are shown in three panels for the ***initial*** assessment. Channels are sorted on the y-axis according to Regions of Interest (ROIs) according to the following nomenclature: LF = Left Frontal, CF = Central Frontal, RF = Right Frontal, LC = Left Central, CC = Central (Vertex), RC = Right Central, LP = Left Parietal, CP = Central Parietal, RP = Right Parietal, LT = Left Temporal, RT = Right Temporal, LO = Left Occipital, CO = Central Occipital, RO = Right Occipital. The frequency bands, delta, theta, alpha, sigma, beta 1, beta 2, gamma 1, and gamma 2 are shown on the x-axis. As clear from the figure, most of the variance found in REM sleep power across frequencies is due to genetic factors.

**Supplementary Figure 2:** In these figures genetic (A), shared environmental (C) and unique environmental/error (E) are shown in three panels for the ***follow-up*** assessment. Channels are sorted on the y-axis according to Regions of Interest (ROIs) according to the following nomenclature: LF = Left Frontal, CF = Central Frontal, RF = Right Frontal, LC = Left Central, CC = Central (Vertex), RC = Right Central, LP = Left Parietal, CP = Central Parietal, RP = Right Parietal, LT = Left Temporal, RT = Right Temporal, LO = Left Occipital, CO = Central Occipital, RO = Right Occipital. The frequency bands, delta, theta, alpha, sigma, beta 1, beta 2, gamma 1, and gamma 2 are shown on the x-axis. Similar to the initial assessment, most of the variance found in REM sleep power across frequencies is due to genetic factors.

**Supplementary Table 1:** The amount of variance explained by genes (i.e., latent factor A) for all 58 channels and 8 frequency bands: delta (1 – 4.6 Hz), theta (4.8 – 7.8 Hz), alpha (8 – 10.8 Hz), sigma (11 – 16 Hz), beta 1 (16.2 – 20 Hz), beta 2 (20.2 – 24 Hz), gamma 1 (24.2 – 34 Hz) and gamma 2 (34.2 – 44 Hz).

**Supplementary Table 2:** The amount of variance explained by genes (i.e., latent factor A) for all 58 channels in the delta band is shown in the second column. The third and fourth column depict the limits of the corresponding 95% confidence interval, while the Akaike information criteria (ACI) for the ACE model and the saturated model are shown in the fifth and sixth column respectively. We found no statistically significant difference between AIC values for the full or saturated model (t(57) = -0.57; p = 0.57). Note that when the values for A (genetic contribution) are very low or zero, then the lower bound of the confidence interval is not calculated and is denoted with a dash (-).

**Supplementary Table 3:** The amount of variance explained by genes (i.e., latent factor A) for all 58 channels in the theta band is shown in the second column. The third and fourth column depict the limits of the corresponding 95% confidence interval, while the Akaike information criteria (ACI) for the ACE model and the saturated model are shown in the fifth and sixth column respectively. We found no statistically significant difference between AIC values for the full or saturated model (t(57) = -1.37; p = 0.18). Note that when the values for A (genetic contribution) are very low or zero, then the lower bound of the confidence interval is not calculated and is denoted with a dash (-).

**Supplementary Table 4:** The amount of variance explained by genes (i.e., latent factor A) for all 58 channels in the alpha band is shown in the second column. The third and fourth column depict the limits of the corresponding 95% confidence interval, while the Akaike information criteria (ACI) for the ACE model and the saturated model are shown in the fifth and sixth column respectively. We found no statistically significant difference between AIC values for the full or saturated model (t(57) = -1.38; p = 0.17). Note that when the values for A (genetic contribution) are very low or zero, then the lower bound of the confidence interval is not calculated and is denoted with a dash (-).

**Supplementary Table 5:** The amount of variance explained by genes (i.e., latent factor A) for all 58 channels in the sigma band is shown in the second column. The third and fourth column depict the limits of the corresponding 95% confidence interval, while the Akaike information criteria (ACI) for the ACE model and the saturated model are shown in the fifth and sixth column respectively. We found no statistically significant difference between AIC values for the full or saturated model (t(57) = 0.75; p = 0.45). Note that when the values for A (genetic contribution) are very low or zero, then the lower bound of the confidence interval is not calculated and is denoted with a dash (-).

**Supplementary Table 6:** The amount of variance explained by genes (i.e., latent factor A) for all 58 channels in the beta 1 band is shown in the second column. The third and fourth column depict the limits of the corresponding 95% confidence interval, while the Akaike information criteria (ACI) for the ACE model and the saturated model are shown in the fifth and sixth column respectively. We found no statistically significant difference between AIC values for the full or saturated model (t(57) = -0.95; p = 0.34). Note that when the values for A (genetic contribution) are very low or zero, then the lower bound of the confidence interval is not calculated and is denoted with a dash (-).

**Supplementary Table 7:** The amount of variance explained by genes (i.e., latent factor A) for all 58 channels in the beta 2 band is shown in the second column. The third and fourth column depict the limits of the corresponding 95% confidence interval, while the Akaike information criteria (ACI) for the ACE model and the saturated model are shown in the fifth and sixth column respectively. We found no statistically significant difference between AIC values for the full or saturated model (t(57) = -0.06; p = 0.95). Note that when the values for A (genetic contribution) are very low or zero, then the lower bound of the confidence interval is not calculated and is denoted with a dash (-).

**Supplementary Table 8:** The amount of variance explained by genes (i.e., latent factor A) for all 58 channels in the gamma 1 band is shown in the second column. The third and fourth column depict the limits of the corresponding 95% confidence interval, while the Akaike information criteria (ACI) for the ACE model and the saturated model are shown in the fifth and sixth column respectively. We found no statistically significant difference between AIC values for the full or saturated model (t(57) = 2.28; p = 0.03). Note that when the values for A (genetic contribution) are very low or zero, then the lower bound of the confidence interval is not calculated and is denoted with a dash (-).

**Supplementary Table 9:** The amount of variance explained by genes (i.e., latent factor A) for all 58 channels in the gamma 2 band is shown in the second column. The third and fourth column depict the limits of the corresponding 95% confidence interval, while the Akaike information criteria (ACI) for the ACE model and the saturated model are shown in the fifth and sixth column respectively. We found no statistically significant difference between AIC values for the full or saturated model (t(57) = 0.44; p = 0.66).

**Supplementary Table 1**

| **Channel** | **Delta** | **Theta** | **Alpha** | **Sigma** | **Beta 1** | **Beta 2** | **Gamma 1** | **Gamma 2** |
| --- | --- | --- | --- | --- | --- | --- | --- | --- |
| 1 | 0 | 0.92 | 0.38 | 0.27 | 0.93 | 0.59 | 0.80 | 0.77 |
| 2 | 0.60 | 0.31 | 0.95 | 0.54 | 0.93 | 0.91 | 0.94 | 0.96 |
| 3 | 0 | 0.66 | 0.49 | 0.59 | 0.93 | 0.72 | 0.92 | 0.90 |
| 4 | 0.68 | 0.39 | 0.78 | 0.26 | 0.91 | 0.85 | 0.89 | 0.62 |
| 5 | 0 | 0.97 | 0.44 | 0.48 | 0.86 | 0.51 | 0.76 | 0.91 |
| 6 | 0.72 | 0.61 | 0.59 | 0.64 | 0.92 | 0.89 | 0.94 | 0.89 |
| 7 | 0.36 | 0.08 | 0.93 | 0.31 | 0.93 | 0.97 | 0.92 | 0.80 |
| 8 | 0 | 0.91 | 0.42 | 0.62 | 0.94 | 0.71 | 0.91 | 0.90 |
| 9 | 0 | 0.95 | 0.54 | 0.47 | 0.90 | 0.78 | 0.95 | 0.82 |
| 10 | 0 | 0.89 | 0.20 | 0.59 | 0.91 | 0.85 | 0.92 | 0.92 |
| 11 | 0.53 | 0.63 | 0.66 | 0.74 | 0.87 | 0.87 | 0.90 | 0.84 |
| 12 | 0 | 0.86 | 0.52 | 0.43 | 0.88 | 0.79 | 0.89 | 0.81 |
| 13 | 0.26 | 0.25 | 0.52 | 0.60 | 0.89 | 0.94 | 0.96 | 0.90 |
| 14 | 0.32 | 0.17 | 0.79 | 0.40 | 0.82 | 0.81 | 0.86 | 0.99 |
| 15 | 0.16 | 0.87 | 0.73 | 0.42 | 0.92 | 0.94 | 0.96 | 0.57 |
| 16 | 0.90 | 0.74 | 0.65 | 0.60 | 0.79 | 0.67 | 0.49 | 0.74 |
| 17 | 0.80 | 0.55 | 0.13 | 0.59 | 0.58 | 0.74 | 0.67 | 0.59 |
| 18 | 0.86 | 0.63 | 0.70 | 0.51 | 0.78 | 0.81 | 0.80 | 0.84 |
| 19 | 0.55 | 0.79 | 0.49 | 0.65 | 0.90 | 0.91 | 0.88 | 0.83 |
| 20 | 0.82 | 0 | 0.55 | 0 | 0 | 0.88 | 0.71 | 0 |
| 21 | 0 | 0.92 | 0.57 | 0.23 | 0.09 | 0.77 | 0.20 | 0.14 |
| 22 | 0 | 0.80 | 0.58 | 0 | 0 | 0.91 | 0.90 | 0.51 |
| 23 | 0.50 | 0.64 | 0.50 | 0.26 | 0.47 | 0.89 | 0.88 | 0.73 |
| 24 | 0.67 | 0.79 | 0.31 | 0 | 0 | 0.90 | 0.95 | 0.84 |
| 25 | 0.12 | 0.87 | 0.52 | 0.55 | 0.89 | 0.88 | 0.89 | 0.84 |
| 27 | 0.04 | 0.74 | 0.76 | 0.89 | 0.89 | 0.86 | 0.89 | 0.80 |
| 28 | 0.37 | 0.57 | 0.13 | 0.87 | 0.78 | 0.91 | 0.99 | 1 |
| 29 | 0.68 | 0.95 | 0.24 | 0.35 | 0.67 | 0.83 | 0.81 | 0.58 |
| 30 | 0.31 | 0.91 | 0.27 | 0.92 | 0.91 | 0.85 | 0.80 | 0.73 |
| 31 | 0.07 | 0.84 | 0.07 | 0.89 | 0.86 | 0.84 | 0.67 | 0.56 |
| 32 | 0 | 0.81 | 0.03 | 0.90 | 0.89 | 0.87 | 0.74 | 0.59 |
| 33 | 0 | 0.65 | 0.10 | 0.82 | 0.84 | 0.72 | 0.65 | 0.51 |
| 34 | 0.06 | 0.56 | 0 | 0.24 | 0.75 | 0.82 | 0.78 | 0.74 |
| 35 | 0.55 | 0.94 | 0.54 | 0.82 | 0.86 | 0.84 | 0.81 | 0.64 |
| 36 | 0.39 | 0.88 | 0.59 | 0.78 | 0.73 | 0.59 | 0.49 | 0.34 |
| 37 | 0.55 | 0.81 | 0.33 | 0.89 | 0.93 | 0.87 | 0.78 | 0.33 |
| 38 | 0.25 | 0.82 | 0.56 | 0.91 | 0.85 | 0.80 | 0.54 | 0.37 |
| 39 | 0.14 | 0 | 0.35 | 0.82 | 0.79 | 0.80 | 0.76 | 0.67 |
| 40 | 0.38 | 0.89 | 0.38 | 0.70 | 0.76 | 0.76 | 0.76 | 0.56 |
| 41 | 0.60 | 0.90 | 0.80 | 0.87 | 0.87 | 0.88 | 0.87 | 0.90 |
| 42 | 0.82 | 0.92 | 0.89 | 0.89 | 0.93 | 0.95 | 0.95 | 0.89 |
| 43 | 0.38 | 0.89 | 0.61 | 0.86 | 0.81 | 0.87 | 0.65 | 0.61 |
| 44 | 0 | 0.55 | 0 | 0.10 | 0.54 | 0.69 | 0.71 | 0.61 |
| 45 | 0.21 | 0.79 | 0.48 | 0.84 | 0.85 | 0.87 | 0.75 | 0.70 |
| 46 | 0.84 | 0.92 | 0.90 | 0.92 | 0.94 | 0.94 | 0.91 | 0.80 |
| 47 | 0.82 | 0.95 | 0.86 | 0.27 | 0.44 | 0.86 | 0.75 | 0.60 |
| 48 | 0.54 | 0.64 | 0.91 | 0.91 | 0.93 | 0.90 | 0.90 | 0.99 |
| 49 | 0.25 | 0.80 | 0.03 | 0.11 | 0.17 | 0.65 | 0.66 | 0.62 |
| 50 | 0.81 | 0.95 | 0.96 | 0.62 | 0.96 | 0.93 | 0.93 | 0.77 |
| 51 | 0.84 | 0.67 | 0.95 | 0.91 | 0.84 | 0.74 | 0.94 | 0.01 |
| 52 | 0.86 | 0.96 | 0.79 | 0.10 | 0.31 | 0.85 | 0.80 | 0 |
| 53 | 0.79 | 0.40 | 0.91 | 0.90 | 0.83 | 0.71 | 0.70 | 0.77 |
| 54 | 0.56 | 0.16 | 0.91 | 0.23 | 0.93 | 0.96 | 0.96 | 0.99 |
| 55 | 0.61 | 0.94 | 0.71 | 0.39 | 0.87 | 0.64 | 0.70 | 0 |
| 56 | 0.57 | 0.94 | 0.46 | 0.05 | 0.12 | 0.61 | 0 | 0 |
| 57 | 0.65 | 0.12 | 0.71 | 0.35 | 0.91 | 0.62 | 0.81 | 0.71 |
| 58 | 0.57 | 0.12 | 0.69 | 0.31 | 0.91 | 0.79 | 0.83 | 0.68 |

**Supplementary Table 2**

| **Channel** | **A** | **CI lower** | **CI upper** | **AIC** | **AIC saturated** |
| --- | --- | --- | --- | --- | --- |
| 1 | 0 | - | 0.5339 | 424.7000 | 424.0800 |
| 2 | 0.5989 | 0.1076 | 0.9410 | 466.4700 | 471.4000 |
| 3 | 0 | - | 0.6756 | 449.7800 | 446.0900 |
| 4 | 0.6797 | 0.3546 | 0.8471 | 432.4100 | 438.0200 |
| 5 | 0 | - | 0.4044 | 386.3800 | 385.8600 |
| 6 | 0.7222 | 0.0435 | 0.9135 | 426.8600 | 431.2400 |
| 7 | 0.3646 | 0 | 0.8851 | 457.5200 | 463.3100 |
| 8 | 0 | - | 0.8144 | 400.1100 | 402.3600 |
| 9 | 0 | - | 0.8296 | 399.2500 | 402.4100 |
| 10 | 0 | - | 0.6865 | 398.0100 | 403.6600 |
| 11 | 0.5289 | 0 | 0.8928 | 418.3000 | 423.5700 |
| 12 | 0 | - | 0.5107 | 453.3900 | 445.1100 |
| 13 | 0.2591 | 0 | 0.8399 | 421.2900 | 428.8100 |
| 14 | 0.3237 | 0 | 0.9138 | 422.1900 | 431.8600 |
| 15 | 0.1591 | - | 0.7869 | 443.1400 | 448 |
| 16 | 0.8999 | 0.7777 | 0.9524 | 389.7000 | 399.7800 |
| 17 | 0.7987 | 0.5610 | 0.9055 | 383.4500 | 390.8900 |
| 18 | 0.8601 | 0.6773 | 0.9352 | 372.2600 | 377.7900 |
| 19 | 0.5476 | 0 | 0.9099 | 365.8900 | 366.1100 |
| 20 | 0.8186 | 0.6016 | 0.9145 | 379.3300 | 387.6200 |
| 21 | 0 | - | 0.6537 | 300.7700 | 302.6100 |
| 22 | 0 | - | 0.7673 | 352.3400 | 359.5700 |
| 23 | 0.5047 | 0 | 0.8681 | 385.1900 | 391.3300 |
| 24 | 0.6730 | 0.0944 | 0.9266 | 374.5800 | 384.0100 |
| 25 | 0.1226 | - | 0.8460 | 361.6300 | 368.7200 |
| 26 | 0.4184 | 0 | 0.9232 | 183.5800 | 117.2500 |
| 27 | 0.0362 | - | 0.8211 | 397.0900 | 397.5100 |
| 28 | 0.3693 | 0 | 0.8782 | 379.2700 | 386.0800 |
| 29 | 0.6761 | 0.1109 | 0.9560 | 349.9000 | 361.1000 |
| 30 | 0.3109 | 0 | 0.8784 | 470.0600 | 470.6000 |
| 31 | 0.0675 | - | 0.8107 | 486.5500 | 483.1200 |
| 32 | 0 | - | 0.6984 | 502.0900 | 469 |
| 33 | 0 | - | 0.7506 | 444.7300 | 433.0500 |
| 34 | 0.0592 | - | 0.7982 | 488.0400 | 485.8800 |
| 35 | 0.5484 | 0.0999 | 0.9506 | 548.9800 | 554.2700 |
| 36 | 0.3882 | 0 | 0.9338 | 560.9800 | 563.8700 |
| 37 | 0.5488 | 0 | 0.8828 | 380.5500 | 389.0900 |
| 38 | 0.2537 | 0 | 0.9176 | 517.2100 | 511.6300 |
| 39 | 0.1429 | - | 0.8602 | 392.5700 | 396.8500 |
| 40 | 0.3843 | 0.0424 | 0.9508 | 575.7400 | 577.0500 |
| 41 | 0.5997 | 0.2772 | 0.7939 | 392.1800 | 388.6300 |
| 42 | 0.8207 | 0.6087 | 0.9154 | 393.7000 | 402.0100 |
| 43 | 0.3798 | 0.0461 | 0.9555 | 484.8400 | 483.6600 |
| 44 | 0 | - | 0.6812 | 509.3300 | 500.1800 |
| 45 | 0.2137 | - | 0.9046 | 507.9300 | 502.0500 |
| 46 | 0.8394 | 0.6295 | 0.9266 | 359.3800 | 366.4900 |
| 47 | 0.8203 | 0.1530 | 0.9208 | 388.0400 | 397.8400 |
| 48 | 0.5424 | 0 | 0.9030 | 357.2700 | 362.9500 |
| 49 | 0.2542 | - | 0.8873 | 411.8700 | 402.6100 |
| 50 | 0.8091 | 0.5809 | 0.9105 | 334.3900 | 336.4700 |
| 51 | 0.8360 | 0.5821 | 0.9301 | 386.8800 | 387.4400 |
| 52 | 0.8630 | 0.6425 | 0.9395 | 425.1100 | 407.8900 |
| 53 | 0.7935 | 0.5663 | 0.9007 | 404.6500 | 411.4600 |
| 54 | 0.5593 | 0 | 0.9129 | 464.2600 | 467.5700 |
| 55 | 0.6111 | 0.2424 | 0.8123 | 348.8100 | 357.4800 |
| 56 | 0.5688 | 0.1569 | 0.7992 | 327.4300 | 337.8200 |
| 57 | 0.6497 | 0 | 0.8612 | 428.8100 | 431.1900 |
| 58 | 0.5745 | 0 | 0.8616 | 441.2400 | 445.0700 |

**Supplementary Table 3**

| **Channel** | **A** | **CI lower** | **CI upper** | **AIC** | **AIC saturated** |
| --- | --- | --- | --- | --- | --- |
| 1 | 0.9224 | 0.8046 | 0.9654 | 196.5600 | 191.0200 |
| 2 | 0.3093 | 0 | 0.9192 | 289.5700 | 288.8600 |
| 3 | 0.6622 | 0.1164 | 0.9329 | 210.3400 | 211.1800 |
| 4 | 0.3890 | 0 | 0.8292 | 259.0600 | 263.4200 |
| 5 | 0.9677 | 0.9095 | 0.9864 | 184.3100 | 172.8400 |
| 6 | 0.6081 | 0 | 0.8756 | 231.7400 | 235.5800 |
| 7 | 0.0823 | - | 0.8345 | 266.9500 | 270.5400 |
| 8 | 0.9087 | 0.7766 | 0.9589 | 194.9000 | 188.0600 |
| 9 | 0.9536 | 0.8741 | 0.9800 | 208.6400 | 192.8300 |
| 10 | 0.8892 | 0.3429 | 0.9516 | 182.9600 | 183.0600 |
| 11 | 0.6255 | 0.0492 | 0.9256 | 221.7500 | 227.1800 |
| 12 | 0.8613 | 0.3082 | 0.9399 | 200.2200 | 193.4900 |
| 13 | 0.2460 | - | 0.8671 | 220.8000 | 224.7700 |
| 14 | 0.1651 | - | 0.8709 | 246.3000 | 252.0200 |
| 15 | 0.8731 | 0.3346 | 0.9648 | 184.7300 | 181.4800 |
| 16 | 0.7425 | 0.2025 | 0.9462 | 204.0600 | 205.0300 |
| 17 | 0.5470 | 0.0076 | 0.9268 | 235.2900 | 229.8500 |
| 18 | 0.6338 | 0 | 0.8965 | 173.3200 | 175.5500 |
| 19 | 0.7926 | 0.2782 | 0.9649 | 184.1600 | 173.6000 |
| 20 | 0 | - | 0.1303 | 178.7000 | 176.4200 |
| 21 | 0.9206 | 0.4731 | 0.9668 | 138.6000 | 140.6200 |
| 22 | 0.7997 | 0.2852 | 0.9697 | 175.4700 | 178.6600 |
| 23 | 0.6410 | 0.1704 | 0.9506 | 205.9200 | 206.9700 |
| 24 | 0.7927 | 0.3230 | 0.9784 | 206.2500 | 210.9800 |
| 25 | 0.8704 | 0.3489 | 0.9746 | 189.9900 | 186.5200 |
| 26 | 0.9248 | 0 | 0.9864 | 118.7300 | 120.4500 |
| 27 | 0.7432 | 0.2530 | 0.9619 | 204.4700 | 211.0300 |
| 28 | 0.5750 | 0.0821 | 0.9398 | 215.3500 | 214.7500 |
| 29 | 0.9494 | 0.8489 | 0.9794 | 230.1600 | 223.2500 |
| 30 | 0.9118 | 0.7891 | 0.9593 | 293.1400 | 298.8200 |
| 31 | 0.8423 | 0.6296 | 0.9278 | 294.6500 | 303.6800 |
| 32 | 0.8090 | 0.5147 | 0.9172 | 266.0900 | 271.8100 |
| 33 | 0.6528 | 0 | 0.8926 | 225.8600 | 231.5000 |
| 34 | 0.5623 | 0.0495 | 0.9273 | 299.7500 | 302.7200 |
| 35 | 0.9384 | 0.8545 | 0.9713 | 318.8500 | 326.9700 |
| 36 | 0.8831 | 0.7267 | 0.9458 | 312.8500 | 322.0600 |
| 37 | 0.8146 | 0.2022 | 0.9339 | 215.9000 | 220.6800 |
| 38 | 0.8222 | 0.6037 | 0.9169 | 280.6800 | 290.8800 |
| 39 | 0 | - | 0.7903 | 248.3900 | 250.3900 |
| 40 | 0.8925 | 0.7483 | 0.9501 | 332.0700 | 341.4800 |
| 41 | 0.8997 | 0.3274 | 0.9527 | 197.7100 | 203.5100 |
| 42 | 0.9222 | 0.4561 | 0.9636 | 208.2000 | 209.0200 |
| 43 | 0.8894 | 0.7534 | 0.9477 | 258.5200 | 268.5700 |
| 44 | 0.5510 | 0 | 0.8842 | 303.6100 | 303.1600 |
| 45 | 0.7940 | 0.2527 | 0.9568 | 270.2900 | 280.6000 |
| 46 | 0.9156 | 0.8002 | 0.9613 | 197.1700 | 199.8000 |
| 47 | 0.9473 | 0.8788 | 0.9752 | 216.6200 | 219.6400 |
| 48 | 0.6350 | 0.0325 | 0.9206 | 187.9000 | 196.0400 |
| 49 | 0.7986 | 0.1597 | 0.9344 | 264.5800 | 261.1600 |
| 50 | 0.9457 | 0.8707 | 0.9748 | 155.0500 | 151.7000 |
| 51 | 0.6663 | 0 | 0.8982 | 235.1200 | 239.6100 |
| 52 | 0.9586 | 0.8999 | 0.9808 | 220.8700 | 215.9900 |
| 53 | 0.4038 | 0 | 0.8932 | 209 | 219.2400 |
| 54 | 0.1592 | - | 0.8734 | 279.1900 | 285.6300 |
| 55 | 0.9367 | 0.8432 | 0.9713 | 156.6700 | 153.4300 |
| 56 | 0.9407 | 0.8263 | 0.9752 | 204.2600 | 176.9400 |
| 57 | 0.1202 | - | 0.8257 | 232.5100 | 237.3200 |
| 58 | 0.1246 | - | 0.8298 | 240.1300 | 244.9300 |

**Supplementary Table 4**

| **Channel** | **A** | **CI lower** | **CI upper** | **AIC** | **AIC saturated** |
| --- | --- | --- | --- | --- | --- |
| 1 | 0.3839 | 0 | 0.8895 | 66.4600 | 68.3600 |
| 2 | 0.9518 | 0.8871 | 0.9774 | 116.0700 | 123.4600 |
| 3 | 0.4851 | 0 | 0.9142 | 74.4700 | 78.1800 |
| 4 | 0.7765 | 0.0658 | 0.8972 | 100.9700 | 108.6300 |
| 5 | 0.4365 | 0 | 0.9258 | 70.8800 | 73.3900 |
| 6 | 0.5919 | 0 | 0.9105 | 81.4500 | 86.2900 |
| 7 | 0.9278 | 0.3607 | 0.9664 | 88.5400 | 94.9200 |
| 8 | 0.4201 | 0 | 0.9026 | 72.9200 | 74.0100 |
| 9 | 0.5380 | 0 | 0.9247 | 75.2500 | 75.8600 |
| 10 | 0.2045 | - | 0.8940 | 62.0900 | 64.0200 |
| 11 | 0.6635 | 0.2212 | 0.9624 | 63.4600 | 70.6100 |
| 12 | 0.5185 | 0 | 0.9114 | 74.7800 | 80.3100 |
| 13 | 0.5237 | 0.0284 | 0.9274 | 69.4400 | 76.2600 |
| 14 | 0.7883 | 0.2712 | 0.9642 | 91.2300 | 98.1500 |
| 15 | 0.7260 | 0.2391 | 0.9568 | 51.5500 | 55.0800 |
| 16 | 0.6542 | 0.2051 | 0.9607 | 64.8900 | 63.3800 |
| 17 | 0.1333 | - | 0.8158 | 103.6700 | 96.6300 |
| 18 | 0.7012 | 0.2392 | 0.9646 | 27.6800 | 35.0800 |
| 19 | 0.4899 | 0.1788 | 0.9790 | 65.0400 | 71.7700 |
| 20 | 0.5496 | 0.1565 | 0.9675 | 38.6300 | 46.7400 |
| 21 | 0.5716 | 0.1298 | 0.9656 | 31.2500 | 34.7600 |
| 22 | 0.5778 | 0.1543 | 0.9691 | 43.4900 | 46.2800 |
| 23 | 0.5044 | 0.0044 | 0.9243 | 100.8500 | 108.2900 |
| 24 | 0.3083 | 0 | 0.9010 | 98.2900 | 105.0300 |
| 25 | 0.5156 | 0 | 0.9188 | 110.1200 | 98.5300 |
| 26 | 0.3420 | - | 0.9930 | 68.5600 | -11.4800 |
| 27 | 0.7642 | 0 | 0.8846 | 140.4100 | 141.0700 |
| 28 | 0.1284 | - | 0.8678 | 135.0200 | 118.9600 |
| 29 | 0.2389 | - | 0.8935 | 137.8300 | 136.0800 |
| 30 | 0.2693 | 0 | 0.8384 | 225.7500 | 235.4700 |
| 31 | 0.0662 | - | 0.7255 | 246.0700 | 253.4900 |
| 32 | 0.0250 | - | 0.7934 | 221.3300 | 228.8900 |
| 33 | 0.1037 | - | 0.7289 | 180.5700 | 184.1900 |
| 34 | 0 | - | 0.3448 | 220.3700 | 225.4800 |
| 35 | 0.5384 | 0.1495 | 0.9547 | 272.5900 | 272.0900 |
| 36 | 0.5866 | 0 | 0.9043 | 271.2000 | 277.5300 |
| 37 | 0.3267 | 0 | 0.8886 | 116.2800 | 106.6100 |
| 38 | 0.5567 | - | 0.8591 | 251.2900 | 257.6900 |
| 39 | 0.3468 | 0 | 0.9221 | 101.8000 | 99.6200 |
| 40 | 0.3768 | 0 | 0.9097 | 286.6000 | 292.3800 |
| 41 | 0.7952 | 0.5493 | 0.9043 | 133.4000 | 138.8400 |
| 42 | 0.8904 | 0.7411 | 0.9494 | 123.0800 | 124.6200 |
| 43 | 0.6098 | 0.1409 | 0.9432 | 212.5300 | 220.8600 |
| 44 | 0 | - | 0.6231 | 240.3300 | 236.8800 |
| 45 | 0.4812 | 0 | 0.9250 | 209.8000 | 214.9300 |
| 46 | 0.9022 | 0.7675 | 0.9553 | 101.3400 | 104.6000 |
| 47 | 0.8629 | 0.6917 | 0.9356 | 105.5800 | 108.2900 |
| 48 | 0.9146 | 0.8061 | 0.9598 | 83.9100 | 89.9600 |
| 49 | 0.0267 | - | 0.8040 | 157.3200 | 157.0900 |
| 50 | 0.9584 | 0.9024 | 0.9805 | 29.8400 | 35.7700 |
| 51 | 0.9536 | 0.8714 | 0.9803 | 85.9200 | 90.8000 |
| 52 | 0.7936 | 0.2638 | 0.9612 | 48.6400 | 54.7000 |
| 53 | 0.9111 | 0.4208 | 0.9583 | 69.4200 | 77.3100 |
| 54 | 0.9074 | 0.7864 | 0.9566 | 104.0400 | 112.7400 |
| 55 | 0.7121 | 0.1915 | 0.9475 | 31.3300 | 39.1200 |
| 56 | 0.4611 | 0 | 0.9301 | 56.8500 | 64.3600 |
| 57 | 0.7083 | 0.0798 | 0.9212 | 75.7800 | 83.6300 |
| 58 | 0.6895 | 0.1416 | 0.9349 | 76.4700 | 85.2500 |

**Supplementary Table 5**

| **Channel** | **A** | **CI lower** | **CI upper** | **AIC** | **AIC saturated** |
| --- | --- | --- | --- | --- | --- |
| 1 | 0.2739 | 0 | 0.9258 | 5.1600 | 9.8200 |
| 2 | 0.5426 | 0.1385 | 0.9651 | 64.3400 | 61.8500 |
| 3 | 0.5924 | 0.1481 | 0.9538 | -5.1700 | 0.8700 |
| 4 | 0.2648 | - | 0.8946 | 76.5300 | 64.5000 |
| 5 | 0.4768 | 0.1662 | 0.9852 | -13.8200 | -5.7200 |
| 6 | 0.6359 | 0.0308 | 0.9239 | 21.1200 | 24.3500 |
| 7 | 0.3143 | 0.0302 | 0.9528 | 58.1000 | 56.8800 |
| 8 | 0.6233 | 0.1982 | 0.9718 | -17.6000 | -9.3000 |
| 9 | 0.4695 | 0.1511 | 0.9808 | -2.5400 | -0.4700 |
| 10 | 0.5941 | 0.1906 | 0.9754 | -13.8800 | -5.4700 |
| 11 | 0.7438 | 0.1940 | 0.9551 | 19.4200 | 19.2400 |
| 12 | 0.4330 | 0.0453 | 0.9524 | -2.9600 | 5.6600 |
| 13 | 0.6021 | 0.1190 | 0.9467 | 14.7400 | 15.9100 |
| 14 | 0.4031 | 0.0266 | 0.9468 | 37.5600 | 38.8500 |
| 15 | 0.4226 | 0.1515 | 0.9834 | -25.1000 | -17 |
| 16 | 0.6016 | 0.0847 | 0.9422 | 25.4300 | 29.2100 |
| 17 | 0.5935 | 0 | 0.9163 | -1.0400 | -0.5000 |
| 18 | 0.5113 | 0.1525 | 0.9717 | -9.9800 | -10.0400 |
| 19 | 0.6496 | 0.1707 | 0.9538 | -13.2500 | -7.3400 |
| 20 | 0 | - | 0.2665 | 51.7000 | 30.1100 |
| 21 | 0.2291 | 0 | 0.9073 | 4.3300 | -7.4700 |
| 22 | 0 | - | 0.3716 | 75.9500 | 51.1200 |
| 23 | 0.2600 | 0 | 0.8726 | 35.0400 | 35.2600 |
| 24 | 0 | - | 0.6128 | 82.2900 | 63.3800 |
| 25 | 0.5479 | 0.0482 | 0.9355 | -6.3600 | -5.9800 |
| 26 | 0.7658 | 0 | 0.9687 | 13.9500 | -1.9400 |
| 27 | 0.8914 | 0.7589 | 0.9484 | 9.5700 | 13.3900 |
| 28 | 0.8673 | 0.2610 | 0.9387 | -5.6800 | -2.2500 |
| 29 | 0.3507 | - | 0.9358 | 63.4100 | 65.5000 |
| 30 | 0.9177 | 0.7964 | 0.9625 | 97.2400 | 94.4800 |
| 31 | 0.8897 | 0.7053 | 0.9514 | 95.1200 | 90.9600 |
| 32 | 0.8979 | 0.6905 | 0.9574 | 75.9000 | 67.6600 |
| 33 | 0.8206 | 0.3037 | 0.9208 | 34.0300 | 37.8300 |
| 34 | 0.2427 | 0 | 0.8844 | 72.3200 | 78.1100 |
| 35 | 0.8164 | 0.5390 | 0.9194 | 128.3300 | 128.9100 |
| 36 | 0.7752 | 0.3714 | 0.9075 | 126.1900 | 119 |
| 37 | 0.8912 | 0.3610 | 0.9495 | -13.5000 | -12.3700 |
| 38 | 0.9060 | 0.7536 | 0.9581 | 74.3000 | 69.3700 |
| 39 | 0.8155 | 0.2062 | 0.9389 | -4.4400 | -2.3700 |
| 40 | 0.6987 | 0.3054 | 0.8691 | 145.2200 | 141.7800 |
| 41 | 0.8722 | 0.3869 | 0.9406 | 31.3900 | 29.6900 |
| 42 | 0.8864 | 0.4549 | 0.9472 | 25.2600 | 25.5600 |
| 43 | 0.8649 | 0.6936 | 0.9370 | 76.1500 | 73.8600 |
| 44 | 0.1042 | - | 0.7626 | 96.6900 | 103.0600 |
| 45 | 0.8380 | 0.6436 | 0.9236 | 89.4600 | 85.0600 |
| 46 | 0.9227 | 0.8261 | 0.9636 | 29.8200 | 32.0900 |
| 47 | 0.2673 | 0.0430 | 0.9592 | 54.1100 | 45.2400 |
| 48 | 0.9119 | 0.3065 | 0.9582 | -7.5700 | -0.8500 |
| 49 | 0.1066 | - | 0.8616 | 77.8300 | 80.6100 |
| 50 | 0.6233 | 0.1833 | 0.9705 | -17.2300 | -13.8500 |
| 51 | 0.9102 | 0.7787 | 0.9604 | 41.2000 | 43.3300 |
| 52 | 0.1045 | - | 0.7022 | 50.6900 | 47.4100 |
| 53 | 0.9007 | 0.7780 | 0.9529 | 26.7800 | 34.7800 |
| 54 | 0.2275 | - | 0.9209 | 87.6700 | 80.1100 |
| 55 | 0.3916 | 0 | 0.9011 | 6.6700 | 9.7700 |
| 56 | 0.0535 | - | 0.9090 | 43.8900 | 41.9100 |
| 57 | 0.3495 | 0 | 0.8760 | 26.1000 | 27.2000 |
| 58 | 0.3112 | 0 | 0.8672 | 35.6700 | 33.6800 |

**Supplementary Table 6**

| **Channel** | **A** | **CI lower** | **CI upper** | **AIC** | **AIC saturated** |  |
| --- | --- | --- | --- | --- | --- | --- |
| 1 | 0.9262 | 0.4177 | 0.9665 | -45.9300 | -41.0200 | |
| 2 | 0.9275 | 0.6970 | 0.9674 | -63.7300 | -69.9500 | |
| 3 | 0.9295 | 0.6095 | 0.9674 | -70.5400 | -62.3900 | |
| 4 | 0.9148 | 0.6990 | 0.9642 | -24.5800 | -31.9000 | |
| 5 | 0.8585 | 0.3636 | 0.9405 | -35.4500 | -27.5800 | |
| 6 | 0.9187 | 0.6827 | 0.9636 | -61.3200 | -60.3200 | |
| 7 | 0.9349 | 0.6888 | 0.9704 | -46.7600 | -51.4300 | |
| 8 | 0.9367 | 0.6190 | 0.9716 | -86.5800 | -78.2300 | |
| 9 | 0.9023 | 0.5174 | 0.9581 | -68.0600 | -60.0600 | |
| 10 | 0.9067 | 0.4889 | 0.9599 | -67.1100 | -58.2500 | |
| 11 | 0.8669 | 0.5743 | 0.9431 | -12.7400 | -20.2200 | |
| 12 | 0.8760 | 0.4946 | 0.9473 | -46.8800 | -39.4600 | |
| 13 | 0.8865 | 0.6328 | 0.9504 | -15.4600 | -16.8100 | |
| 14 | 0.8173 | 0.3803 | 0.9193 | -50.9400 | -58.4000 | |
| 15 | 0.9231 | 0.5101 | 0.9639 | -37.9200 | -33.4000 | |
| 16 | 0.7933 | 0.2533 | 0.9013 | -27.1800 | -31.2800 | |
| 17 | 0.5816 | 0 | 0.9012 | -117.4600 | -121.6600 | |
| 18 | 0.7775 | 0.1612 | 0.9511 | -46.1300 | -43.2700 | |
| 19 | 0.8980 | 0.2841 | 0.9688 | -125.4100 | 293.0900 | |
| 20 | 0 | - | 0.7704 | -40.8000 | -59.4500 | |
| 21 | 0.0945 | 0 | 0.8820 | -73.6500 | -78.9200 | |
| 22 | 0 | - | 0.7690 | -20.1800 | -43.0400 | |
| 23 | 0.4699 | 0 | 0.8824 | -78.5100 | -76.3100 | |
| 24 | 0 | - | 0.8091 | -39.5300 | -57.2400 | |
| 25 | 0.8900 | 0.2861 | 0.9478 | -115.9200 | -118.2100 | |
| 26 | 0.5526 | 0 | 0.9677 | -39.5900 | -52.1500 | |
| 27 | 0.8944 | 0.4957 | 0.9507 | -106.2400 | -107.2300 | |
| 28 | 0.7755 | 0.2962 | 0.9033 | -95.9600 | -100.6100 | |
| 29 | 0.6688 | 0.0028 | 0.9464 | -52.9300 | -46.6200 | |
| 30 | 0.9124 | 0.5700 | 0.9594 | -60.5100 | -56.3200 | |
| 31 | 0.8646 | 0.5497 | 0.9393 | -58.3000 | -53.8200 | |
| 32 | 0.8920 | 0.5819 | 0.9546 | -70.2700 | -67.9400 | |
| 33 | 0.8398 | 0.4323 | 0.9300 | -106.5700 | -98.5600 | |
| 34 | 0.7486 | 0.1451 | 0.9349 | -81.3100 | -74.6300 | |
| 35 | 0.8634 | 0.5749 | 0.9407 | -29.2700 | -29.7500 | |
| 36 | 0.7309 | 0.2092 | 0.8922 | -40.1200 | -39.0800 | |
| 37 | 0.9253 | 0.4892 | 0.9655 | -144.1200 | -135.3500 | |
| 38 | 0.8505 | 0.5431 | 0.9360 | -79.7600 | -79.5500 | |
| 39 | 0.7927 | 0.1670 | 0.9401 | -134.6200 | -130.3600 | |
| 40 | 0.7646 | 0.3119 | 0.8998 | -19.8300 | -17.8400 | |
| 41 | 0.8748 | 0.4775 | 0.9421 | -97.8200 | -97.2800 | |
| 42 | 0.9278 | 0.5486 | 0.9663 | -110.0500 | -103 | |
| 43 | 0.8083 | 0.5793 | 0.9106 | -60.9600 | -61.0200 | |
| 44 | 0.5390 | 0 | 0.8235 | -52.2100 | -46.4200 | |
| 45 | 0.8458 | 0.3900 | 0.9287 | -54.4100 | -57.0700 | |
| 46 | 0.9351 | 0.4550 | 0.9699 | -84.5200 | -81.4100 | |
| 47 | 0.4393 | 0.1026 | 0.9648 | -72.5500 | -77.1900 | |
| 48 | 0.9328 | 0.3879 | 0.9682 | -117.5900 | -110.3600 | |
| 49 | 0.1715 | 0 | 0.8677 | -55.2200 | -55.0600 | |
| 50 | 0.9630 | 0.3593 | 0.9824 | -132.8600 | -131.0200 | |
| 51 | 0.8374 | 0.3754 | 0.9315 | -44.8200 | -52.9600 | |
| 52 | 0.3132 | 0.0030 | 0.9449 | -75.7500 | -73.7400 | |
| 53 | 0.8312 | 0.3771 | 0.9205 | -33.8800 | -36.1700 | |
| 54 | 0.9265 | 0.6719 | 0.9668 | -36.6900 | -46.3700 | |
| 55 | 0.8719 | 0.3126 | 0.9401 | -64.1300 | -61.1900 | |
| 56 | 0.1201 | 0 | 0.9150 | -57.0400 | -53.2200 | |
| 57 | 0.9100 | 0.6220 | 0.9598 | -31.0600 | -29.9900 | |
| 58 | 0.9107 | 0.6490 | 0.9600 | -27.6600 | -35.1100 | |

**Supplementary Table 7**

| **Channel** | **A** | **CI lower** | **CI upper** | **AIC** | **AIC saturated** |
| --- | --- | --- | --- | --- | --- |
| 1 | 0.5864 | 0 | 0.8049 | -50.4400 | -52.6500 |
| 2 | 0.9058 | 0.6324 | 0.9577 | -125.2800 | -129.0600 |
| 3 | 0.7239 | 0.2059 | 0.8731 | -96.9000 | -96.7000 |
| 4 | 0.8511 | 0.4805 | 0.9408 | -93.9100 | -93.1100 |
| 5 | 0.5134 | 0 | 0.7592 | -43.2000 | -44.5600 |
| 6 | 0.8934 | 0.6411 | 0.9530 | -141.7000 | -139.0200 |
| 7 | 0.9726 | 0.7575 | 0.9873 | -121.7900 | -122.8100 |
| 8 | 0.7097 | 0.0807 | 0.8657 | -116.1400 | -113.2000 |
| 9 | 0.7762 | 0.2806 | 0.9060 | -122.4300 | -122.0600 |
| 10 | 0.8521 | 0.3725 | 0.9380 | -100.7000 | -96.6700 |
| 11 | 0.8677 | 0.5669 | 0.9448 | -69.5200 | -83.1100 |
| 12 | 0.7867 | 0.3129 | 0.9115 | -72.7800 | -77.1900 |
| 13 | 0.9378 | 0.7500 | 0.9725 | -53.6800 | -67.4100 |
| 14 | 0.8075 | 0.3957 | 0.9204 | -114.0800 | -113.9700 |
| 15 | 0.9421 | 0.7278 | 0.9739 | -59.2700 | -66.1000 |
| 16 | 0.6744 | 0.1145 | 0.8708 | -74.5200 | -76.8100 |
| 17 | 0.7448 | 0 | 0.8770 | -190.0500 | -184.8800 |
| 18 | 0.8054 | 0.4333 | 0.9169 | -94.0300 | -93.2700 |
| 19 | 0.9120 | 0.4116 | 0.9585 | -191.8900 | -183.8900 |
| 20 | 0.8799 | 0.5478 | 0.9459 | -163.5300 | -158.7800 |
| 21 | 0.7668 | 0.1983 | 0.9820 | -174.0500 | -164.8500 |
| 22 | 0.9062 | 0.5744 | 0.9581 | -168.5400 | -163.6400 |
| 23 | 0.8917 | 0.3663 | 0.9486 | -176.2800 | -170.2000 |
| 24 | 0.8998 | 0.7713 | 0.9541 | -184.3400 | -177.1300 |
| 25 | 0.8811 | 0.4340 | 0.9449 | -187.4900 | -196.1400 |
| 26 | 0.3730 | 0 | 0.9488 | -58.3800 | -89.5800 |
| 27 | 0.8630 | 0.5372 | 0.9383 | -181.1300 | -192.1600 |
| 28 | 0.9062 | 0.6694 | 0.9607 | -177.6900 | -187.1700 |
| 29 | 0.8326 | 0.2145 | 0.9374 | -129.6400 | -121.2900 |
| 30 | 0.8540 | 0.5543 | 0.9366 | -150.3700 | -154.3900 |
| 31 | 0.8364 | 0.6930 | 0.9306 | -145.8400 | -147.2500 |
| 32 | 0.8671 | 0.5314 | 0.9456 | -147.8100 | -146.2000 |
| 33 | 0.7213 | 0.0708 | 0.8770 | -186.9600 | -178.6000 |
| 34 | 0.8200 | 0.2535 | 0.9149 | -162.2000 | -155.3100 |
| 35 | 0.8445 | 0.5144 | 0.9351 | -112.9700 | -120.1900 |
| 36 | 0.5865 | 0 | 0.8319 | -135.3100 | -130.7400 |
| 37 | 0.8685 | 0.4621 | 0.9406 | -212.0900 | -206.9100 |
| 38 | 0.8009 | 0.4067 | 0.9177 | -171.3300 | -169.6200 |
| 39 | 0.7987 | 0.1710 | 0.9051 | -204.4400 | -197.0500 |
| 40 | 0.7626 | 0.2978 | 0.9012 | -113.7800 | -110.5700 |
| 41 | 0.8833 | 0.5443 | 0.9469 | -182.5600 | -180.4100 |
| 42 | 0.9549 | 0.6543 | 0.9791 | -195.0500 | -188.4800 |
| 43 | 0.8698 | 0.5860 | 0.9424 | -152.9300 | -151.4700 |
| 44 | 0.6904 | 0 | 0.8599 | -141.1400 | -136.9500 |
| 45 | 0.8745 | 0.5833 | 0.9448 | -144.8500 | -145.9800 |
| 46 | 0.9376 | 0.6197 | 0.9718 | -167.0700 | -163.6700 |
| 47 | 0.8574 | 0.4235 | 0.9339 | -172.6500 | -168.6200 |
| 48 | 0.8963 | 0.4509 | 0.9517 | -184.6900 | -176.5800 |
| 49 | 0.6503 | 0 | 0.8383 | -129.6500 | -132.9000 |
| 50 | 0.9331 | 0.6347 | 0.9692 | -205.2200 | -203.4400 |
| 51 | 0.7372 | 0.1694 | 0.8979 | -109.2500 | -122.7600 |
| 52 | 0.8474 | 0.4267 | 0.9301 | -176 | -168.7200 |
| 53 | 0.7087 | 0.2255 | 0.8750 | -102.2300 | -100.0400 |
| 54 | 0.9556 | 0.7050 | 0.9796 | -120.7700 | -121.5000 |
| 55 | 0.6422 | 0.0321 | 0.8480 | -116.3200 | -111.5800 |
| 56 | 0.6125 | 0 | 0.8454 | -126.4800 | -123.5800 |
| 57 | 0.6164 | 0 | 0.8524 | -48.5300 | -52.0800 |
| 58 | 0.7942 | 0.3566 | 0.9160 | -63.9800 | -69.4500 |

**Supplementary Table 8**

| **Channel** | **A** | **CI lower** | **CI upper** | **AIC** | **AIC saturated** |
| --- | --- | --- | --- | --- | --- |
| 1 | 0.8028 | 0.2347 | 0.9122 | -109.4800 | -112.8200 |
| 2 | 0.9438 | 0.6905 | 0.9744 | -142.0400 | -143.9500 |
| 3 | 0.9157 | 0.6035 | 0.9615 | -155.4400 | -157.6900 |
| 4 | 0.8868 | 0.6240 | 0.9536 | -133.1000 | -136.8600 |
| 5 | 0.7639 | 0.0722 | 0.9030 | -102.2100 | -97.8200 |
| 6 | 0.9429 | 0.7122 | 0.9741 | -175.9800 | -177.3300 |
| 7 | 0.9191 | 0.6757 | 0.9636 | -150.9600 | -154.2900 |
| 8 | 0.9112 | 0.6307 | 0.9615 | -156.8700 | -156.6300 |
| 9 | 0.9523 | 0.6874 | 0.9796 | -156.4800 | -160.2100 |
| 10 | 0.9207 | 0.4186 | 0.9655 | -150 | -147.1000 |
| 11 | 0.8952 | 0.6125 | 0.9540 | -134.2000 | -142.0400 |
| 12 | 0.8875 | 0.5385 | 0.9526 | -135.7600 | -133.1500 |
| 13 | 0.9571 | 0.7356 | 0.9804 | -136.3000 | -140.4500 |
| 14 | 0.8560 | 0.3595 | 0.9434 | -88.9900 | -117.1300 |
| 15 | 0.9597 | 0.6117 | 0.9812 | -147.2200 | -145.9900 |
| 16 | 0.4888 | 0 | 0.7317 | -99.1300 | -106.6300 |
| 17 | 0.6725 | 0 | 0.8442 | -186.8300 | -178.7400 |
| 18 | 0.7956 | 0.3439 | 0.9068 | -126.0400 | -126.2300 |
| 19 | 0.8765 | 0.4310 | 0.9425 | -184.6000 | -179.9300 |
| 20 | 0.7051 | 0.0716 | 0.8685 | -174.6500 | -171.1000 |
| 21 | 0.2033 | 0.0157 | 0.9754 | -181.2200 | -177.8200 |
| 22 | 0.8953 | 0.8549 | 0.9538 | -173.7600 | -172.0400 |
| 23 | 0.8812 | 0.4920 | 0.9452 | -174.4800 | -171.3200 |
| 24 | 0.9468 | 0.7089 | 0.9758 | -195.5900 | -197.0300 |
| 25 | 0.8865 | 0.5555 | 0.9492 | -182.8200 | -193.0900 |
| 26 | 0 | - | 0.9096 | -54.9900 | -75.1400 |
| 27 | 0.8879 | 0.6390 | 0.9507 | -178.7500 | -188.5000 |
| 28 | 0.9912 | 0.8201 | 0.9960 | -72.5600 | -145.0200 |
| 29 | 0.8121 | 0.1334 | 0.9290 | -117.7500 | -111.9300 |
| 30 | 0.8022 | 0.4281 | 0.9180 | -122.2700 | -129.4900 |
| 31 | 0.6735 | 0.1327 | 0.8726 | -115.9800 | -120.3400 |
| 32 | 0.7408 | 0.2120 | 0.8986 | -122.8700 | -125.4800 |
| 33 | 0.6516 | 0 | 0.8461 | -171.9600 | -167.4900 |
| 34 | 0.7848 | 0.3037 | 0.9027 | -132.1600 | -136.7100 |
| 35 | 0.8085 | 0.3590 | 0.9245 | -86.4300 | -98.7800 |
| 36 | 0.4903 | 0 | 0.8031 | -118 | -119.5900 |
| 37 | 0.7754 | 0.2863 | 0.9014 | -190.1600 | -185.7000 |
| 38 | 0.5367 | 0 | 0.7977 | -140.8200 | -140.0600 |
| 39 | 0.7594 | 0.1035 | 0.8892 | -196.3500 | -190.4900 |
| 40 | 0.7621 | 0.2357 | 0.9095 | -87.4500 | -94.7500 |
| 41 | 0.8702 | 0.5607 | 0.9425 | -175.8200 | -173.5200 |
| 42 | 0.9477 | 0.6787 | 0.9760 | -190.2600 | -187.4500 |
| 43 | 0.6549 | 0.0933 | 0.8520 | -110.4300 | -109.8500 |
| 44 | 0.7100 | 0.1657 | 0.8792 | -118.2900 | -124.8700 |
| 45 | 0.7536 | 0.2916 | 0.8958 | -103.2400 | -107.3600 |
| 46 | 0.9133 | 0.6446 | 0.9620 | -154.6800 | -155.3100 |
| 47 | 0.7486 | 0.2355 | 0.8908 | -159.6800 | -163.6000 |
| 48 | 0.8986 | 0.5590 | 0.9541 | -169.6700 | -167.7800 |
| 49 | 0.6559 | 0 | 0.8436 | -118.2500 | -136.4400 |
| 50 | 0.9340 | 0.6308 | 0.9697 | -198.3100 | -197.9600 |
| 51 | 0.9377 | 0.4434 | 0.9738 | -105.3700 | -122.9000 |
| 52 | 0.7985 | 0.1952 | 0.9063 | -172.2900 | -170.3600 |
| 53 | 0.7030 | 0.1893 | 0.8791 | -120.9800 | -116.4800 |
| 54 | 0.9638 | 0.7312 | 0.9833 | -145.9200 | -149.6200 |
| 55 | 0.6983 | 0.1355 | 0.8670 | -142.9400 | -135.7600 |
| 56 | 0 | 0 | 0.7863 | -148.5300 | -149.3800 |
| 57 | 0.8146 | 0.3540 | 0.9186 | -109.8700 | -112.0700 |
| 58 | 0.8304 | 0.4096 | 0.9243 | -121.8100 | -121.4400 |

**Supplementary Table 9**

| **Channel** | **A** | **CI lower** | **CI upper** | **AIC** | **AIC saturated** |
| --- | --- | --- | --- | --- | --- |
| 1 | 0.7749 | 0.0578 | 0.9013 | -237.3400 | -244.8000 |
| 2 | 0.9588 | 0.7795 | 0.9814 | -227.8500 | -242.9600 |
| 3 | 0.8955 | 0.5797 | 0.9533 | -265.4600 | -276.2200 |
| 4 | 0.6238 | 0.0399 | 0.8671 | -235.6300 | -238.6400 |
| 5 | 0.9126 | 0.4843 | 0.9633 | -202.7100 | -214.6200 |
| 6 | 0.8907 | 0.6812 | 0.9522 | -284.4000 | -288.1500 |
| 7 | 0.8016 | 0.3222 | 0.9082 | -271.9300 | -273.4600 |
| 8 | 0.9048 | 0.6579 | 0.9600 | -253.4600 | -261.0700 |
| 9 | 0.8195 | 0.2043 | 0.9227 | -209.3700 | -229.6300 |
| 10 | 0.9228 | 0.5514 | 0.9671 | -255.9800 | -266.9500 |
| 11 | 0.8405 | 0.5109 | 0.9325 | -254.4300 | -266.9100 |
| 12 | 0.8134 | 0.1509 | 0.9196 | -218.2300 | -225.2300 |
| 13 | 0.8976 | 0.6481 | 0.9549 | -267.9000 | -281.4200 |
| 14 | 0.9947 | 0.8314 | 0.9976 | -9.5200 | -154.5600 |
| 15 | 0.5730 | 0.0488 | 0.9260 | -265.7200 | -273.4500 |
| 16 | 0.7415 | 0.2364 | 0.8792 | -266.5900 | -264 |
| 17 | 0.5851 | 0 | 0.7893 | -273.4400 | -269.8900 |
| 18 | 0.8416 | 0.4959 | 0.9321 | -262.5400 | -270.9400 |
| 19 | 0.8320 | 0.1688 | 0.9203 | -259.1300 | -253.4800 |
| 20 | 0 | 0 | 0.4920 | -239.6700 | -239.2000 |
| 21 | 0.1394 | 0 | 0.9351 | -265.4800 | -258.8800 |
| 22 | 0.5095 | 0 | 0.7670 | -253.8600 | -253.4700 |
| 23 | 0.7318 | 0.0499 | 0.8704 | -268.5600 | -264.0800 |
| 24 | 0.8356 | 0.4606 | 0.9251 | -309.3900 | -305.7800 |
| 25 | 0.8432 | 0.4510 | 0.9299 | -269.1500 | -277.5000 |
| 26 | 0 | 0 | 0.8665 | -96.2700 | -150.8100 |
| 27 | 0.7987 | 0.4284 | 0.9110 | -272.3300 | -276.9400 |
| 28 | 0.9998 | 0.8336 | 0.9999 | 15.0700 | -179.2600 |
| 29 | 0.5791 | 0 | 0.8371 | -204.1800 | 346.9000 |
| 30 | 0.7322 | 0.2349 | 0.8726 | -217.9400 | -230.3100 |
| 31 | 0.5575 | 0 | 0.7828 | -210.6800 | -214.2100 |
| 32 | 0.5914 | 0 | 0.8131 | -209.3700 | -207.2300 |
| 33 | 0.5099 | 0 | 0.7724 | -256.7200 | -251.5900 |
| 34 | 0.7351 | 0.1627 | 0.8772 | -231.1000 | -235.0200 |
| 35 | 0.6426 | 0.1181 | 0.8581 | -189.5500 | -198.5600 |
| 36 | 0.3379 | 0 | 0.6704 | -218.5800 | -219.1400 |
| 37 | 0.3279 | 0 | 0.7511 | -248.2200 | -247 |
| 38 | 0.3672 | 0 | 0.6757 | -228.5600 | -227.9500 |
| 39 | 0.6746 | 0 | 0.8631 | -264.7700 | -275.1700 |
| 40 | 0.5594 | 0.0186 | 0.8130 | -190.4400 | -193.7300 |
| 41 | 0.8953 | 0.5985 | 0.9530 | -275.1700 | -271.3700 |
| 42 | 0.8862 | 0.5117 | 0.9479 | -286.5800 | -280.3600 |
| 43 | 0.6141 | 0 | 0.8106 | -201.8800 | -201.5600 |
| 44 | 0.6066 | 0 | 0.8151 | -210.1300 | -217.7700 |
| 45 | 0.7022 | 0.1707 | 0.8627 | -192.9600 | -200.3900 |
| 46 | 0.7982 | 0.3582 | 0.9120 | -252.9200 | -247.0400 |
| 47 | 0.6016 | 0 | 0.8081 | -280.3200 | -289.6600 |
| 48 | 0.9918 | 0.8096 | 0.9962 | -122.0900 | -212.7400 |
| 49 | 0.6226 | 0 | 0.8133 | -212.8500 | -229.7000 |
| 50 | 0.7746 | 0.1656 | 0.8946 | -302.3800 | -296.0500 |
| 51 | 0.0123 | 0.0032 | 0.0437 | -70.1300 | -162.2700 |
| 52 | 0 | - | 0.6922 | -274.9900 | -271.1600 |
| 53 | 0.7746 | 0.0895 | 0.9155 | -216.6400 | -234.6600 |
| 54 | 0.9874 | 0.8164 | 0.9941 | -183.2900 | -230.9100 |
| 55 | 0 | 0 | 0.5310 | -264.4600 | -261.4800 |
| 56 | 0 | 0 | 0.5734 | -249.9800 | -266.5700 |
| 57 | 0.7141 | 0 | 0.8696 | -249.1300 | -250.6500 |
| 58 | 0.6828 | 0 | 0.8496 | -261.4800 | -257.2100 |
