# Supplementary figures and images for: Heritability of REM sleep neurophysiology in adolescence
Source: Transl Psychiatry. 2022 Sep 21;12:399. doi: 10.1038/s41398-022-02106-6 (PMC9492899; doi:10.1038/s41398-022-02106-6)

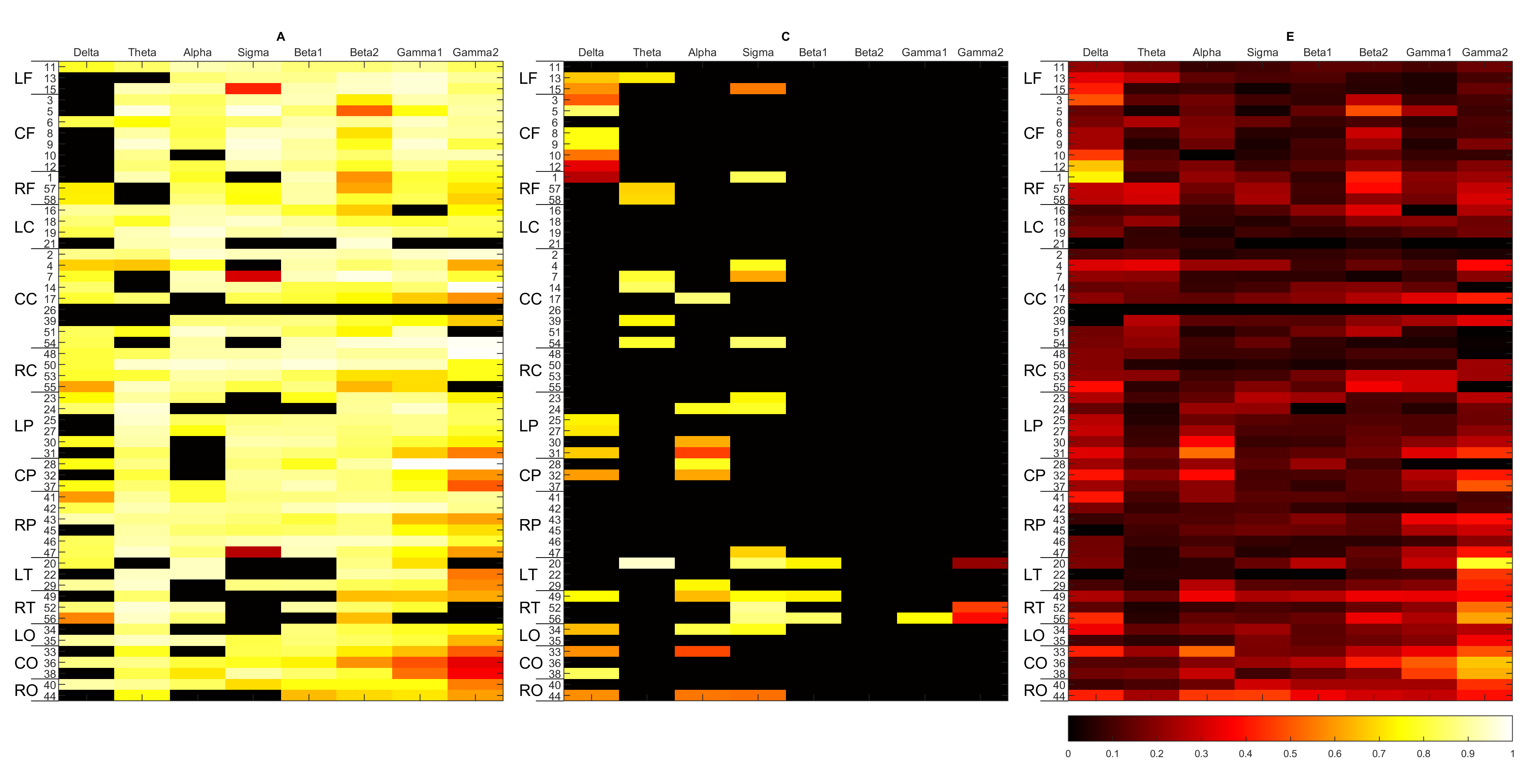

Supplement: Supplementary file 2 — Supplementary Figure 1 [file 41398_2022_2106_MOESM2_ESM.png]

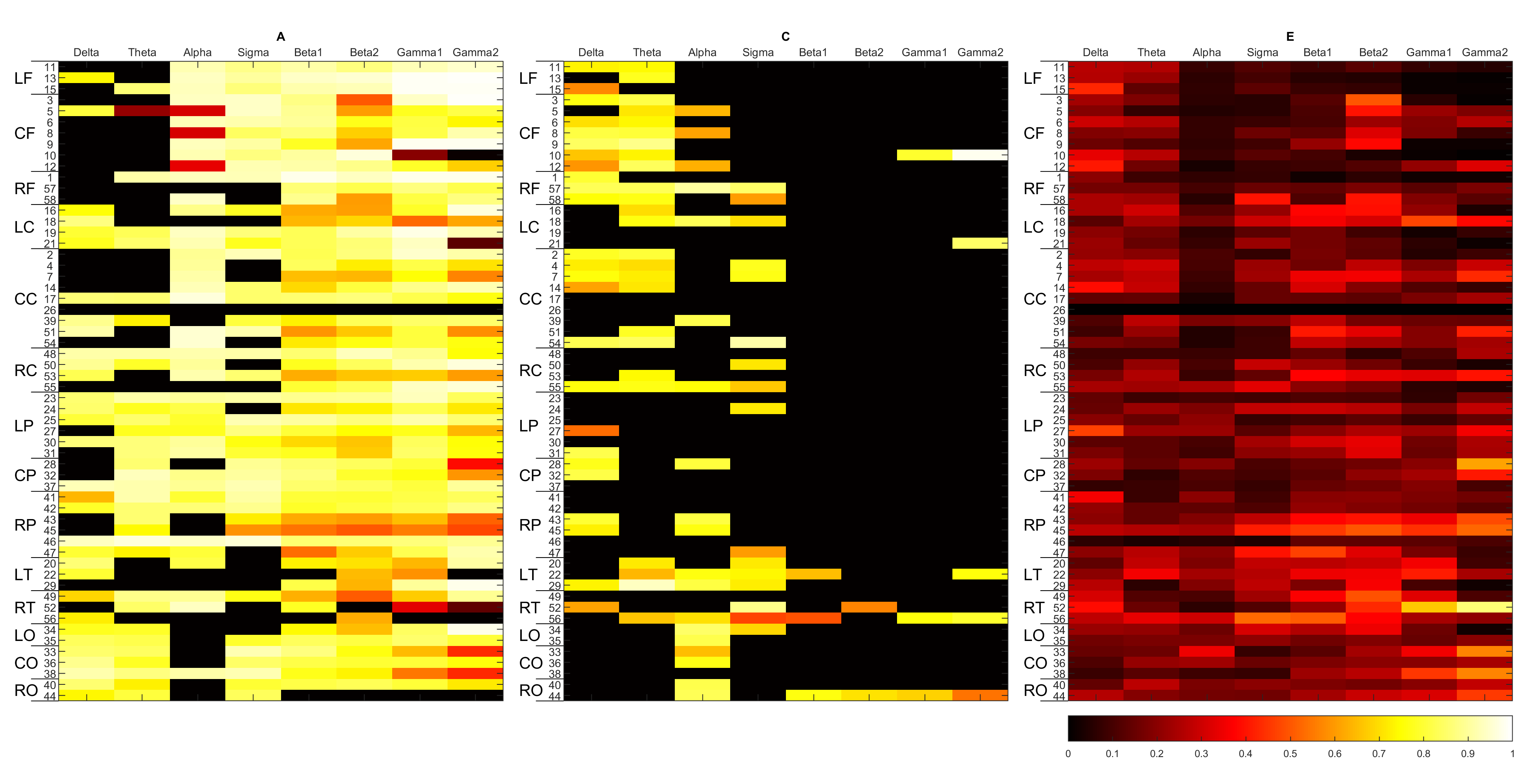

Supplement: Supplementary file 3 — Supplementary Figure 2 [file 41398_2022_2106_MOESM3_ESM.png]
